# Cascade Analysis for Women Presenting With Breast Concerns to a Zonal Hospital in Mwanza, Tanzania

**DOI:** 10.1200/GO.22.00345

**Published:** 2023-03-22

**Authors:** Tara M. Friebel-Klingner, Emma Joo, Matogoro Kirahi, Lydia E. Pace, Elizabeth A. Platz, Nestory Masalu, Leonard Washington, Anne F. Rositch

**Affiliations:** ^1^ Department of Epidemiology, Johns Hopkins Bloomberg School of Public Health, Baltimore, MD; ^2^ Bugando Medical Centre, Mwanza, Tanzania; ^3^ Brigham and Women's Hospital, Boston, MA; ^4^ Sidney Kimmel Comprehensive Cancer Center at Johns Hopkins, Baltimore, MD

## Abstract

**PURPOSE:**

In Tanzania, high breast cancer mortality can be attributed to delays in diagnosis and treatment initiation. We adapted the cascade analysis method to depict sequential steps along the breast cancer care pathway in a tertiary hospital in Mwanza, to identify where correction of loss to attrition would have the biggest impact on improving outcomes.

**METHODS:**

This prospective cohort included adult women presenting with breast concerns between February 2020 and January 2022. Five cascade steps beginning with patients' initial clinical breast assessment (CBA) through cancer treatment were identified: (1) CBA, (2) ordering diagnostic test(s), (3) completion of diagnostic test(s), (4) receipt of final diagnosis, and (5) initiating cancer treatment.

**RESULTS:**

Overall, 721 eligible women with a median age of 42.8 years (IQR, 32.5-55.0) were included. Median time from presentation to treatment initiation was 35 days (IQR, 20-63). For step 1, 39.1% (n = 282) of patients were diagnosed with a benign concern and removed from the cascade. Completion rates for steps 2-4 were 95.0%, 90.2%, and 91.0, respectively. There were 156 (45.6%) patients diagnosed with breast cancer, and for step 5, 71.2% of patients initiated cancer treatment. In steps 2, 3, 4, and 5, there was a loss of 22, 41, 34, and 45 patients, respectively. If loss was eliminated at steps 2, 3, 4, or 5, an additional 6, 12, 11, or 45 patients, respectively, would have completed the pathway.

**CONCLUSION:**

Initiating cancer treatment was identified as the step with the biggest loss and, if remedied, would have the biggest impact on improving breast cancer outcomes at Bugando Medical Centre. These results will inform future programs focused on reducing overall loss in the system and supporting patients with breast cancer.

## INTRODUCTION

Breast cancer is the most common cancer diagnosed in women worldwide, with low- and middle-income countries (LMICs) shouldering a disproportionately higher burden of mortality.^1^ In the past few decades, mortality rates have decreased in high-income countries, but breast cancer incidence and mortality rates in LMICs have dramatically increased.^2^ In the East African lower-middle–income country of Tanzania, it is estimated that 80% of breast cancers are diagnosed at advanced stages.^3,4^ Thus, high breast cancer mortality rates can be attributed, in part, to delayed diagnosis and treatment.^5,6^

**CONTEXT**

**Key Objective**
To our knowledge, this cascade analysis was the first to quantify loss to follow-up and completion rates along the breast cancer care pathway at a Zonal Hospital with comprehensive cancer care in Tanzania.
**Knowledge Generated**
Completion rates for breast cancer diagnostics were high but treatment initiation was lower and represents the step with the greatest loss of patients. These results highlight where in the breast cancer care cascade future resources should be directed to understand the reasons for loss and develop context-specific solutions to achieve high completion rates along the entire cancer care pathway.
**Relevance**
Breast cancer mortality rates in Tanzania are high, like in many low- and middle-income countries. The cascade analysis approach provides direct data-driven feedback to the care team in the health system regarding the overall breast cancer care cascade. Understanding and remediating areas where patients are not completing care are essential to improve breast cancer outcomes, particularly survival.

Bugando Medical Centre (BMC), a tertiary zonal hospital in Mwanza, Tanzania, serves more than 14 million citizens and is one of three tertiary hospitals in the country providing comprehensive cancer care.^4^ Our ongoing Time to A.C.T. study^4,7^ intends to reduce breast cancer morbidity and mortality through standardizing diagnosis and treatment pathways at BMC,^4,8^ informed by the Tanzanian Ministry of Health guidelines^9,10^ and the National Comprehensive Cancer Network cancer care guidelines tailored for sub-Saharan Africa (SSA).^11,12^

In settings like Tanzania where screening mammography is not widely available, experts recommend that health systems prioritize expediting clinical breast assessment (CBA) for symptomatic women.^13^ CBA includes obtaining a history of breast symptoms, a general medical examination, and a clinical breast examination (CBE).^14^ CBA is a critical triage step used to determine appropriate prioritization and is followed by appropriate diagnostic imaging and tissue pathology to determine if a breast concern is malignant or benign. The steps along the cancer care pathway are critical for improving morbidity and mortality from breast cancer from diagnosis to treatment.^4^ Data-driven insight of the breast cancer care pathway at BMC is warranted to understand and identify key steps that, if strengthened, would result in the largest impact on improving breast cancer outcomes.

In 2018, the Lancet Global Health Commission on High-Quality Health Systems recommended that care cascade analyses should be a central component of health systems for understanding the quality of care.^7^ Cascades track the sequential steps on the path toward a successful outcome and are increasingly used to quantitatively assess progress.^8^ Cascades have been used in studies assessing HIV and diabetes and in other LMICs such as Kenya and Mozambique.^8-12^ A cascade analysis is an efficient and effective way to maximize the proportion of patients with successful outcomes within resource-constrained environments, allowing direct feedback for health systems and health care professionals. To date, the patients lost to follow-up along the breast cancer care pathway at BMC have not been quantified.

Thus, for this study, we adapted the cascade analysis^8^ to depict the sequential steps and losses along the breast cancer care pathway of patients presenting to BMC with breast concerns from initial CBA through treatment initiation. By determining and quantifying losses at each step, we aimed to identify specific step(s) along the pathway where the most losses occurred, to inform future efforts to deliver the best guideline-informed breast cancer care for patients at BMC.

## METHODS

### Study Design and Participants

Patients were selected from a prospective cohort of women^4,7^ presenting to BMC with any breast concern(s). For this study, we included any woman older than 18 years who presented from February 2020 through January 2022, with an additional 6 months of follow-up for diagnostic and/or treatment through July 2022. A full-time research associate identified and tracked women with breast concerns through the electronic medical records system, departmental intake registries, and carbon copy interdepartmental referral forms in the departments of oncology, emergency medicine (EM), CBE clinic, surgery outpatient department (SOPD), radiology, and pathology. Tracking forms were placed at the point of entry, typically the EM department, and women were followed with their ID through the hospital system. Our research associate checked daily logs for new patients and patient updates.

At BMC, women with breast concerns generally present to EM or the CBE clinic where a physician performs a CBA. If the breast concern is not clearly benign (ie, mastitis and prescribed antibiotics), the physician commonly orders a breast ultrasound (US). If the US shows a suspicion of breast cancer, then the patient will be referred to the SOPD for diagnostic investigation. At SOPD, appropriate diagnostics tests (eg, fine-needle aspiration cytology [FNA-C], biopsy, or mammogram) will be ordered. If metastatic breast cancer is suspected, a patient may be directly referred to oncology where diagnostic tests would include a chest X-ray, abdominal US, or CT scan. Upon results of the diagnostic test(s), any patient found to have breast cancer would be offered appropriate treatment (surgery and/or chemotherapy-adjuvant, neoadjuvant, or palliative). For patients with a breast cancer diagnosis, we also evaluated the number of breast cancer cases initiating treatment.

### Data Collection

A trained research associate abstracted patient and clinical data from electronic medical records, pathology reports, and paper intake forms from hospital departments. Collected data included (1) patient sociodemographic and health history (age, presenting symptoms, and presenting dates); (2) details of CBA and diagnostic procedures and results; and (3) health outcomes, including final diagnosis and treatment initiation. All data were entered into an electronic database that was encrypted and password protected. This study was reviewed and approved by institutional review boards at the Johns Hopkins Bloomberg School of Public Health, the Catholic University of Health and Allied Sciences, and the Tanzanian National Institute for Medical Research.

### Statistical Analysis

We used the cascade analysis method^15^ to characterize five sequential steps from patient's initial breast consultation through diagnosis and appropriate cancer treatment initiation, henceforth referred to as the breast cancer care cascade. Step 1 included every woman identified with a breast concern who underwent a CBA. The provisional results for the CBA included benign diagnosis, inconclusive, or suspicion of malignancy. After removing the patients with a benign diagnosis, step 2 included patients for whom any diagnostic tests were ordered to further evaluate the concern. Step 3 included patients who completed at least one diagnostic evaluation, and step 4 was defined as the number of patients with a definitive diagnosis. After removing the patients who did not have a breast cancer diagnosis, step 5 included patients who initiated treatment. The cascade analysis quantified the number of patients completing each step and identified the number of patients lost to follow-up at each step.

Furthermore, to determine the step with the largest impact on outcomes, the cascade analysis method quantifies at each step the number of patients who would have been gained back through step 5 if lost to follow-up was eliminated. To quantify this number, a series of conditional probabilities is applied to the absolute number of individuals who did not complete this step. This method assumes that if a patient completed the step at which she was lost to follow-up, she would have also completed the additional downstream steps through step 5^15^ assuming the observed downstream loss to follow-up. Also, it is important to note for patients suspected to have a malignancy or be diagnosed with breast cancer, we would not anticipate reaching 100% completion; thus, we did not depict these as steps and did not calculate gains back in the system at these points in the pathway.

Our main analysis focused on all patients who received CBA for breast; however, during our prospective observational study, the COVID-19 pandemic caused notable delays and disruptions in cancer care across the world, including SSA,^7^ and thus, we evaluated the cascade for 18 months during the primary phase of the COVID-19 pandemic (February 2020-July 2021) and then separately, the subsequent 6 months once the COVID-19 pandemic entered a later phase (August 2021-January 2022) when disruptions to care during the COVID-19 pandemic may have waned.

## RESULTS

Overall, 782 women presented to BMC with breast concerns from February 2020 through January 2022 (Table 1). Forty-one women (5.2%) with an existing breast cancer diagnosis and 20 women (2.6%) younger than 18 years were excluded. The median age of the remaining 721 women was 42.8 years (IQR, 32.5-55.0). Over 24 months of prospective observation, there was an average of 30 women presenting per month (range, 11-45). Almost half (47.2%; n = 340) reported having one of two forms of insurance, MSAMAHA for government employees (10.7%) or the National Health Insurance Fund (36.5%); additionally, approximately half of the women were from the Mwanza region (45.8%; n = 326).

**TABLE 1:**
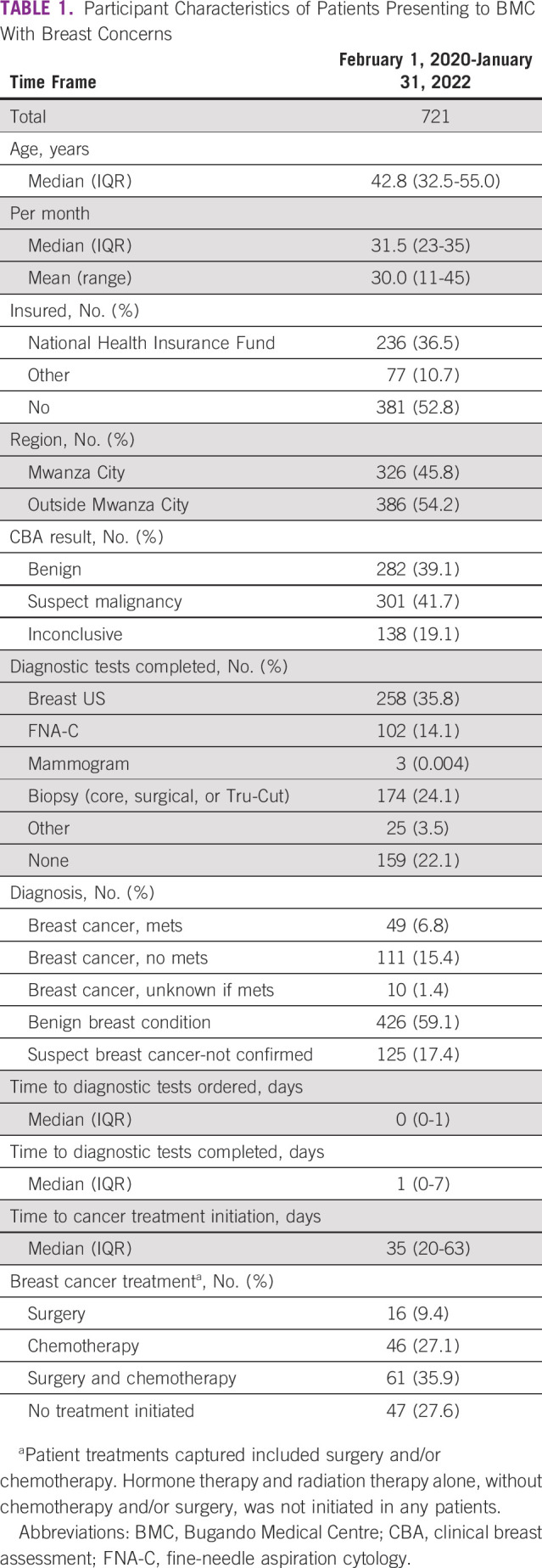
Participant Characteristics of Patients Presenting to BMC With Breast Concerns

Upon presentation to BMC, physician's CBA provisional diagnosis determined 282 (39.1%) benign concerns, 301 (41.7%) were suspected to be malignant, and 138 (19.1%) were deemed inconclusive. Of the total cohort, 562 (77.9%) had at least one diagnostic test completed. Specifically, 258 (35.8%) had a breast US, 102 (14.1%) had an FNA-C, 174 (24.1%) had a tissue biopsy (core needle and/or surgical), 3 (<0.01%) had a mammogram, and 25 (3.5%) had other tests for the investigation of metastasis. For the clinical investigations, 480 (85%) patients had the test ordered the same day and 328 (59%) had at least one test done on the same day. Final diagnosis included 426 (59.1%) benign concerns and 170 (23.6%) breast cancers, of which 49 (28.8%) had metastasis. Median time to cancer treatment initiation was 35 days (IQR, 20-63). Additionally, 125 patients (17.4%) were suspected to have breast cancer, but their diagnosis remained unknown due to incomplete diagnostic tests.

**FIG 1:**
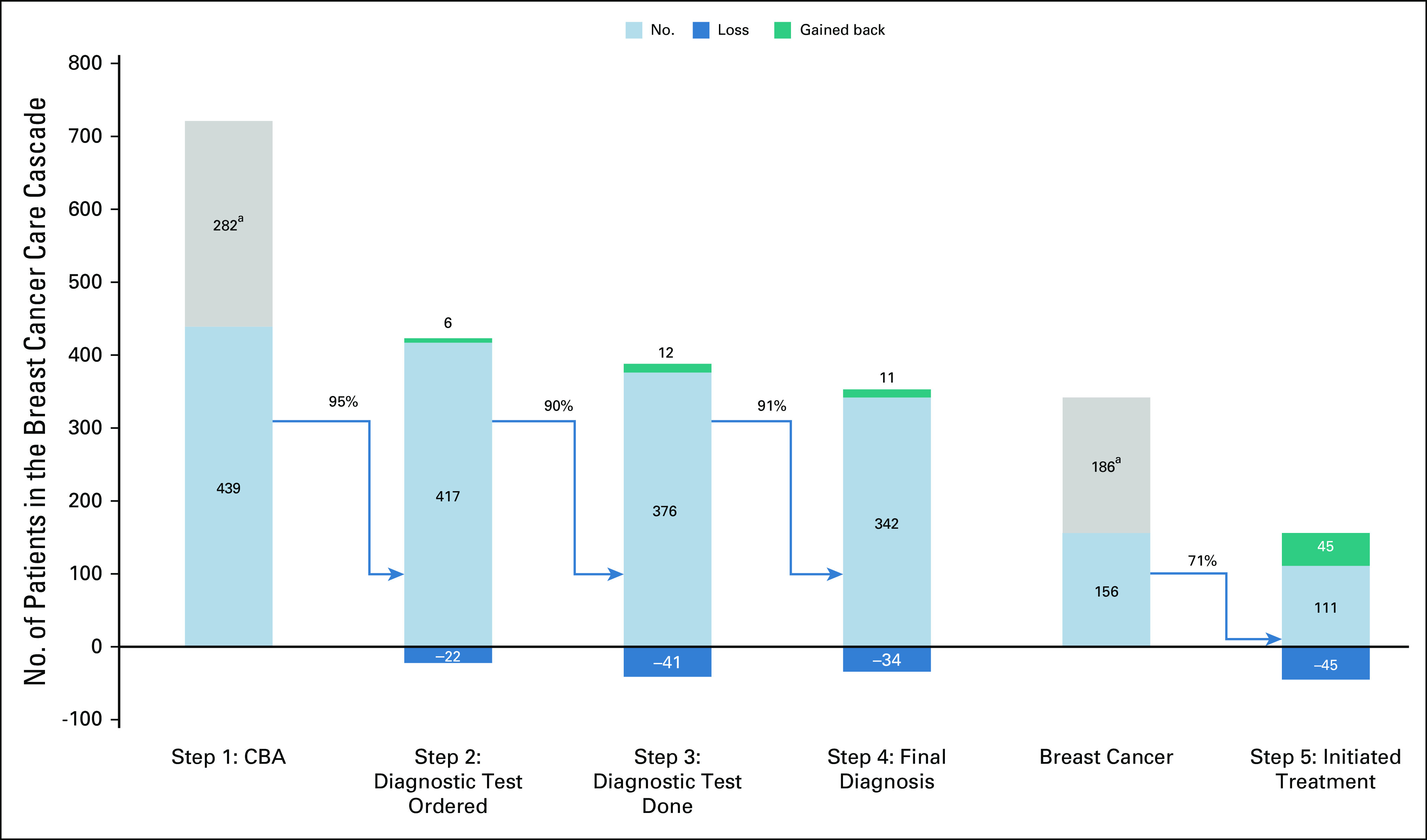
Breast cancer care cascade for patients presenting to BMC with breast concerns, February 2020-January 2022. ^a^Benign patients removed from the cascade. BMC, Bugando Medical Centre.

For the overall breast cancer care cascade (Fig 1 and Table 2), step 1 reflects the 721 CBAs performed, of which 282 (39.1%) were treated as benign and removed from the cascade. For step 2, of the remaining 439 patients that warranted additional tests, 95.0% (n = 417) had at least one diagnostic test ordered. For step 3, 90.2% (n = 376) completed at least one diagnostic test. At step 4, 91.0% (n = 342) of patients received a definitive diagnosis, of whom 41.5% (n = 156) were diagnosed with breast cancer. For step 5, among patients with breast cancer, 71.2% (n = 111) initiated treatment with surgery and/or chemotherapy.

**TABLE 2:**
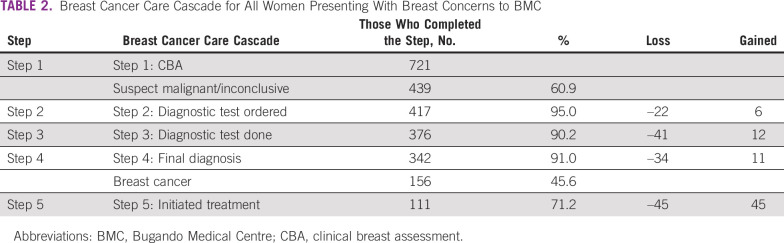
Breast Cancer Care Cascade for All Women Presenting With Breast Concerns to BMC

**FIG A1:**
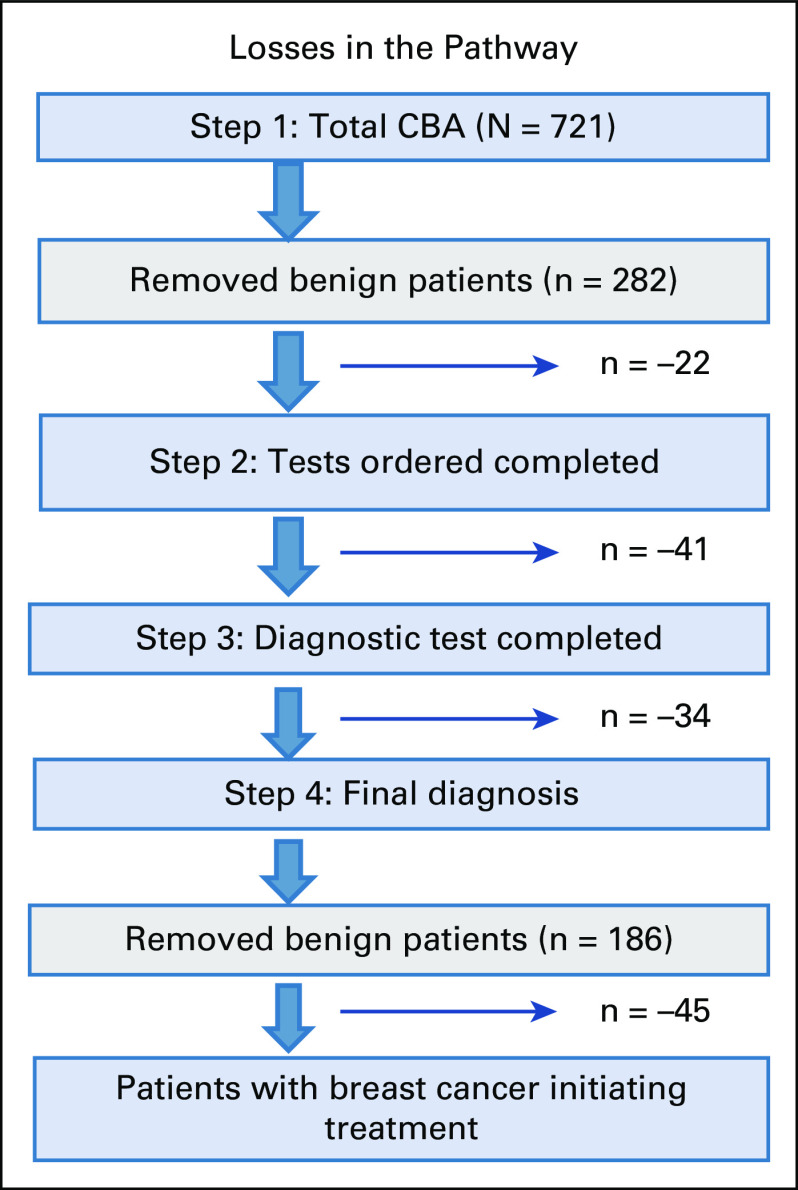
Vertical depiction of losses in the breast cancer care cascade for patients presenting to BMC, February 2020-January 2022. BMC, Bugando Medical Centre; CBA, clinical breast assessment.

In steps 2, 3, and 4, there was a loss of 22, 41, and 34 patients, respectively (Appendix Figure A1). For step 5, there was a loss of 45 patients. If the loss was eliminated at step 2, the pathway would have gained back six patients, assuming the counterfactual that all 22 patients lost at step 2 would have completed all subsequent steps of the pathway through step 5. If the loss was eliminated at step 3, an extra 12 patients would have completed the pathway, and if the loss was eliminated at step 4, 11 additional patients would have completed the pathway. Finally, if the loss was eliminated at step 5, 45 additional patients with breast cancer would have completed the pathway.

Table 3 shows the sensitivity analysis for the early and late COVID-19 cascades (patient characteristics are shown in Appendix Table A1). There were 590 (81.8%) patients in the early COVID-19 period (February 2020-July 2021) and 131 (18.2%) in the late COVID-19 period (August 2021-January 2022). For step 1, of the 590 patients in the early COVID-19 period, 225 (38.1%) were benign and removed. Completion rates for steps 2, 3, and 4 were 94.2% (n = 344), 89.2% (n = 307), and 91.5% (n = 281), respectively. For step 5, of the 122 (43.4%) diagnosed with breast cancer, 86 (69.7%) patients initiated treatment. Thus, at steps 2, 3, 4, and 5, there was a loss of 21, 37, 26, and 37 patients, respectively. For the early COVID-19 period, if the counterfactual had occurred at each step, 5, 10, 8, and 37 patients, respectively, would have been gained back along the pathway. For the 131 patients in the late COVID-19 time period, for step 1, 57 (43.5%) with a benign diagnosis were removed. Completion rates for steps 2, 3, and 4 were 98.6% (n = 72), 94.5% (n = 69), and 88.4% (n = 61), respectively. Thus, at steps 2, 3, 4, and 5, there was a loss of 1, 4, 8, and 8 patients, respectively, and if the counterfactual had occurred, 0, 2, 3, and 8 patients, respectively, would have been gained back along the pathway.

**TABLE 3:**
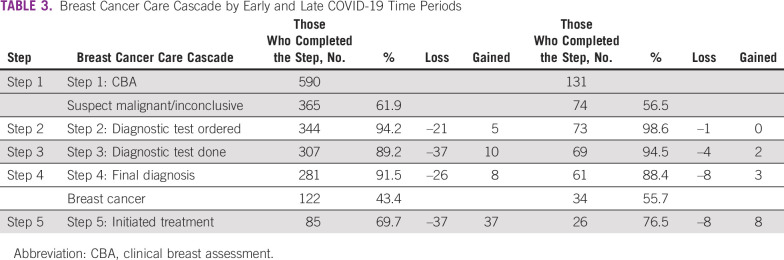
Breast Cancer Care Cascade by Early and Late COVID-19 Time Periods

## DISCUSSION

Understanding the proportion of patients completing steps along the breast cancer care cascade from diagnostics to treatment initiation is imperative to identify patient losses in the system and thus target impactful interventions to improve the breast cancer care at BMC in Mwanza, Tanzania, where breast cancer incidence and mortality continue to rise. For the breast cancer cascade at BMC, the proportion of diagnostic tests ordered and completed was high; however, the proportion of patients initiating cancer treatment reflected the greatest loss of patients. Remedying this step would likely have the biggest impact on improving the breast cancer care pathway at BMC. It is also important to note that in the main analysis, 97 patients were lost to follow-up during steps 2-4, and that 41.5% of patients in the main cascade were diagnosed with breast cancer, potentially missing approximately 40 more breast cancers that went undiagnosed and untreated. Although the biggest loss for each cascade occurred at treatment initiation—and this was true also for the late COVID-19 period—there were more patients initiating cancer treatment during the late period than the early period, potentially signaling a rebound from the negative impacts of the COVID-19 pandemic on health care seeking and systems.

Overall, this cascade analysis study offers descriptive insight into the breast cancer care pathway for a prospective cohort of women seeking care for breast concerns at BMC, a large tertiary zonal hospital in East Africa. A cascade analysis quantifies patient losses at each step of a predefined care pathway, and assuming these losses could be remedied, quantifies the gains back to the system. The step(s) with the greatest gains back are those that a country with limited resources could prioritize for uncovering the explanation(s) for the biggest loss and identifying optimal intervention(s), with the goal of having the biggest impact on improving patient outcomes. In this analysis, steps 2-4, the diagnostic interval, had high rates of completion (>90%), yet treatment initiation among patients diagnosed with breast cancer was lower at 71.2%. Directing resources to understand why almost 30% of patients with pathologically confirmed breast cancer did not initiate potentially life-saving treatment is an essential step toward the goal of improving the breast cancer outcomes in Tanzania.

The literature has noted several health system– and individual-level barriers to initiation of life-saving treatments in other LMICs. Health system–level barriers include the unavailability of essential medicines,^16-20^ staff shortages, and lack of defined national cancer control policies.^21^ Individual-level barriers to treatment could consist of barriers in access, low levels of breast cancer knowledge,^7^ including the inability to pay, lack of transport,^22^ feeling too sick, and stigma, fear, and misconceptions of a cancer diagnosis.^23^ Future research to test interventions and implementation strategies to increase treatment initiation (eg, increase targeted community education and awareness campaigns,^7^ financial assistance, and requiring that WHO essential medicines be available) is a critical next step to improve care and breast cancer outcomes.^21^ In addition, barriers to completion of the care cascade at BMC are currently being studied through the implementation of a new patient navigation program.^23-25^

Given that the cascade analysis is a binary and sequential analysis, it did not allow for patients with a provisional benign diagnosis back the cascade. In our study, of the 282 patients who were given a provisional benign diagnosis and removed from cascade, 14 (5.0%) were ultimately diagnosed with breast cancer. As CBA triage is imperfect, some women deemed 'benign' on CBA are still often sent on for more sensitive diagnostics and thus an underlying cancer could be detected on US or biopsy. If previously determined benign concerns are found to be malignant, then they will be properly diagnosed and offered treatment. Thus, for some disease groups with more complex pathways, nonlinear modifications could improve the applicability of the cascade analysis. For breast cancer, a useful adaptation would be to allow provisional benign patients back in at an appropriate step. Studies have noted similar limitations of the cascade to incorporate nonbinary steps,^15^ including have people move forward or backward, or allowing people who have failed treatment to move back to the beginning of the cascade.^26^

Given the widespread effects of the COVID-19 pandemic, particularly for health systems,^27^ beginning in March 2020 right after our cohort commenced, it seemed prudent to examine the breast cancer care pathway before and after the initial impacts of the COVID-19 pandemic to assess impact on breast cancer care. Although the patient numbers in the late COVID-19 period (August 2021 to January 2022) were relatively small, the proportion of patients completing steps 2 and 3 was slightly higher in the late period, but slightly lower at step 4 compared with the early COVID-19 period. Completion of step 5 (treatment initiation), the step with the biggest loss overall, tended to be higher in the late period (76.5%) compared with the early period (69.7%).

Despite thorough searching for identification of patients with breast concerns, it is possible that our study may have missed women. However, we would anticipate that they would be missing at random and not be biased with regards to completion of the cascade. Another limitation of this study may be underreporting. Our data did not capture detailed information on hormone therapy and radiation therapy; however, it was confirmed by our clinicians that hormone therapy and radiation therapy alone, without chemotherapy and/or surgery, was not prescribed or initiated in any patients, and thus, we were unlikely to miss any patients initiating treatment outside of chemotherapy and/or surgery. Although every effort was made to capture each patient's steps in the cascade by checking multiple data sources and allowing at least 6 months of follow-up after first presentation, some individual procedures may have been missed. We were unable to follow-up patients outside of BMC and therefore do not know what happened to those lost to follow-up. It is possible that patients received care outside of BMC at another hospital, although other facilities with diagnostic and treatment capabilities are more than 800 km (500) miles away. However, to our knowledge, this is the first study using a real-world prospective cohort to understand individual steps along the breast cancer care cascade at BMC, and likely reflects the situation across many LMIC contexts at tertiary facilities. The results from this study can be easily communicated to the providers at BMC, as it can be with other care-providing facilities, to direct future efforts and resources to ensure patients obtain appropriate diagnostic procedures and initiate treatment.

Although no statistical inferences can be made from this descriptive analysis, our results do provide critical insight into the breast cancer care pathway at BMC and indicate that not all patients are completing all steps, and most importantly, among patients with breast cancer, almost a third are not initiating potentially life-saving or life-prolonging treatment. This cascade analysis can be used by BMC to implement and monitor ongoing health system changes. This analysis provides a method/tool that can be used broadly for rapid feedback to our oncologists and staff and therefore highlights the cancer care step with the greatest loss (in this case treatment initiation) where we can focus attention to attempt to understand why and develop context-specific solutions if possible.

This work can help guide future studies aimed at understanding barriers and facilitators to initiating cancer treatment. The findings from such subsequent studies could inform interventions such as a patient navigation program focused on reducing overall loss in the system and supporting patients diagnosed with cancer to initiate and complete treatment. Routine program evaluation is critical for program improvement, and we expect that this study will provide concrete data to inform improvements for the breast cancer care pathway for patients at BMC in Tanzania.

## Figures and Tables

**TABLE A1:**
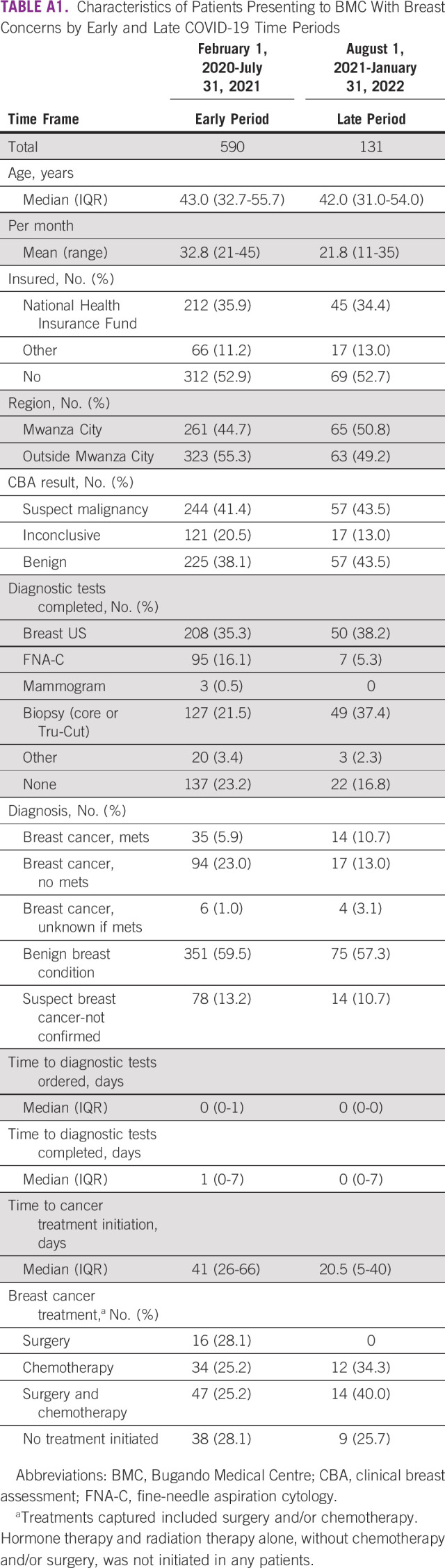
Characteristics of Patients Presenting to BMC With Breast Concerns by Early and Late COVID-19 Time Periods

## References

[1] Sung H Ferlay J Siegel RL et al Global cancer statistics 2020: GLOBOCAN estimates of incidence and mortality worldwide for 36 cancers in 185 countries CA Cancer J Clin 71 209 249 2021 33538338 10.3322/caac.21660

[2] Allemani C Matsuda T Di Carlo V et al Global surveillance of trends in cancer survival 2000-14 (CONCORD-3): Analysis of individual records for 37 513 025 patients diagnosed with one of 18 cancers from 322 population-based registries in 71 countries Lancet 391 1023 1075 2018 29395269 10.1016/S0140-6736(17)33326-3 PMC5879496

[3] Bishop A Dvaladze A Tsu V et al Tanzania Breast Health Care Assessment An Assessment of Breast Cancer Early Detection, Diagnosis, and Treatment in Tanzania. Susan G Komen; Breast Cancer Initiative 2.5, Tanzania Ministry of Health, Community Development, Gender, Elderly, and Children 2017

[4] Sood R Masalu N Connolly RM et al Invasive breast cancer treatment in Tanzania: Landscape assessment to prepare for implementation of standardized treatment guidelines BMC Cancer 21 527 2021 33971839 10.1186/s12885-021-08252-2 PMC8108449

[5] Anderson BO Shyyan R Eniu A et al Breast cancer in limited-resource countries: An overview of the Breast Health Global Initiative 2005 guidelines Breast J 12 S3 S15 2006 16430397 10.1111/j.1075-122X.2006.00199.x

[6] Ginsburg O Yip CH Brooks A et al Breast cancer early detection: A phased approach to implementation Cancer 126 2379 2393 2020 32348566 10.1002/cncr.32887 PMC7237065

[7] Chao CA Huang L Visvanathan K et al Understanding women's perspectives on breast cancer is essential for cancer control: Knowledge, risk awareness, and care-seeking in Mwanza, Tanzania BMC Public Health 20 930 2020 32539723 10.1186/s12889-020-09010-y PMC7296642

[8] Amadori D Serra P Bucchi L et al The Mwanza cancer project Lancet Oncol 17 146 148 2016 26868339 10.1016/S1470-2045(16)00012-7

[9] DeBoer RJ Ndumbalo J Meena S et al Development of a theory-driven implementation strategy for cancer management guidelines in sub-Saharan Africa Implement Sci Commun 1 24 2020 32885183 10.1186/s43058-020-00007-7 PMC7427872

[10] Rositch AF Unger-Saldana K DeBoer RJ et al The role of dissemination and implementation science in global breast cancer control programs: Frameworks, methods, and examples Cancer 126 2394 2404 2020 32348574 10.1002/cncr.32877

[11] Gradishar WJ Moran MS Abraham J et al NCCN Guidelines® insights: Breast cancer, version 4.2021 J Natl Compr Canc Netw 19 484 493 2021 34794122 10.6004/jnccn.2021.0023

[12] Anderson BO NCCN harmonized guidelines for sub-Saharan Africa: A collaborative methodology for translating resource-adapted guidelines into actionable in-country cancer control plans JCO Glob Oncol 6 1419 1421 2020 32970486 10.1200/GO.20.00436 PMC7529522

[13] Echavarria MI Anderson BO Duggan C et al Global uptake of BHGI guidelines for breast cancer Lancet Oncol 15 1421 1423 2014 25456360 10.1016/S1470-2045(14)71102-7

[14] Duggan C Dvaladze A Rositch AF et al The breast health global initiative 2018 global summit on improving breast healthcare through resource-stratified phased implementation: Methods and overview Cancer 126 2339 2352 2020 32348573 10.1002/cncr.32891 PMC7482869

[15] Wagner AD Gimbel S Asbjornsdottir KH et al Cascade analysis: An adaptable implementation strategy across HIV and non-HIV delivery platforms J Acquir Immune Defic Syndr 82 S322 S331 2019 31764270 10.1097/QAI.0000000000002220 PMC6880809

[16] Ewen M Zweekhorst M Regeer B et al Baseline assessment of WHO's target for both availability and affordability of essential medicines to treat non-communicable diseases PLoS One 12 e0171284 2017 28170413 10.1371/journal.pone.0171284 PMC5295694

[17] Laing R Waning B Gray A et al 25 years of the WHO essential medicines lists: Progress and challenges Lancet 361 1723 1729 2003 12767751 10.1016/S0140-6736(03)13375-2

[18] Layer EH Kennedy CE Beckham SW et al Multi-level factors affecting entry into and engagement in the HIV continuum of care in Iringa, Tanzania PLoS One 9 e104961 2014 25119665 10.1371/journal.pone.0104961 PMC4138017

[19] Martei YM Chiyapo S Grover S et al Availability of WHO essential medicines for cancer treatment in Botswana J Glob Oncol 4 1 8 2018 10.1200/JGO.17.00063 PMC6223417 30241225

[20] Martei YM Iwamoto K Barr RD et al Shortages and price variability of essential cytotoxic medicines for treating children with cancers BMJ Glob Health 5 e003282 2020 10.1136/bmjgh-2020-003282 PMC7656942 33173011

[21] Ralefala T Mokokwe L Jammalamadugu S et al Provider barriers and facilitators of breast cancer guideline-concordant therapy delivery in Botswana: A consolidated framework for implementation research analysis Oncologist 26 e2200 e2208 2021 34390287 10.1002/onco.13935 PMC8649035

[22] Teshome B Trabitzsch J Afework T et al Perceived barriers to timely treatment initiation and social support status among women with breast cancer in Ethiopia PLoS One 16 e0257163 2021 34516552 10.1371/journal.pone.0257163 PMC8437283

[23] Chavarri-Guerra Y Soto-Perez-de-Celis E Ramos-Lopez W et al Patient navigation to enhance access to care for underserved patients with a suspicion or diagnosis of cancer Oncologist 24 1195 1200 2019 30498134 10.1634/theoncologist.2018-0133 PMC6738287

[24] Koneru A Jolly PE Blakemore S et al Acceptance of peer navigators to reduce barriers to cervical cancer screening and treatment among women with HIV infection in Tanzania Int J Gynaecol Obstet 138 53 61 2017 28391628 10.1002/ijgo.12174 PMC5482416

[25] Soto-Perez-de-Celis E Chavarri-Guerra Y Ramos-Lopez WA et al Patient navigation to improve early access to supportive care for patients with advanced cancer in resource-limited settings: A randomized controlled trial Oncologist 26 157 164 2021 33210345 10.1002/onco.13599 PMC7873328

[26] Kedziora DJ Abeysuriya R Kerr CC et al The cascade analysis tool: Software to analyze and optimize care cascades Gates Open Res 3 1488 2019 31942536 10.12688/gatesopenres.13031.2 PMC6944813

[27] Martei YM Rick TJ Fadelu T et al Impact of COVID-19 on cancer care delivery in Africa: A cross-sectional survey of oncology providers in Africa JCO Glob Oncol 7 368 377 2021 33689484 10.1200/GO.20.00569 PMC8081536

